# A lung image reconstruction from computed radiography images as a tool to tuberculosis treatment control

**DOI:** 10.1590/1678-9199-JVATITD-1449-18

**Published:** 2019-02-14

**Authors:** Marcela de Oliveira, Sergio Barbosa Duarte, Guilherme Giacomini, Paulo Câmara Marques Pereira, Lenice do Rosário de Souza, José Ricardo de Arruda Miranda, Diana Rodrigues de Pina

**Affiliations:** 1 Universidade Estadual Paulista, Instituto de Biociências de Botucatu, Departamento de Física e Biofísica, Botucatu, SP, Brasil; 2 Centro Brasileiro de Pesquisas Físicas, Departamento de Altas Energias, Rio de Janeiro, RJ, Brasil; 3 Universidade Estadual Paulista, Faculdade de Medicina de Botucatu, Departamento de Doenças Tropicais e Diagnóstico por Imagem, Botucatu, SP, Brasil.

**Keywords:** Tuberculosis, Quantification, Lung involvement, Serum tests, Pulmonary function tests

## Abstract

**Background:**

Background: Tuberculosis (TB) is an infectious lung disease with high worldwide incidence that severely compromises the quality of life in affected individuals. Clinical tests are currently employed to monitor pulmonary status and treatment progression. The present study aimed to apply a three-dimensional (3D) reconstruction method based on chest radiography to quantify lung-involvement volume of TB acute-phase patients before and after treatment. In addition, these results were compared with indices from conventional clinical exams to show the coincidence level.

**Methods:**

A 3D lung reconstruction method using patient chest radiography was applied to quantify lung-involvement volume using retrospective examinations of 50 patients who were diagnosed with pulmonary TB and treated with two different drugs schemes. Twenty-five patients were treated with Scheme I (rifampicin, isoniazid, and pyrazinamide), whereas twenty-five patients were treated with Scheme II (rifampicin, isoniazid, pyrazinamide, and ethambutol). Acute-phase reaction: Serum exams included C-reactive protein levels, erythrocyte sedimentation rate, and albumin levels. Pulmonary function was tested posttreatment.

**Results:**

We found strong agreement between lung involvement and serum indices pre- and posttreatment. Comparison of the functional severity degree with lung involvement based on 3D image quantification for both treatment schemes found a high correlation.

**Conclusions:**

The present 3D reconstruction method produced a satisfactory agreement with the acute-phase reaction, most notably a higher significance level with the C-reactive protein. We also found a quite reasonable coincidence between the 3D reconstruction method and the degree of functional lung impairment posttreatment. The performance of the quantification method was satisfactory when comparing the two treatment schemes. Thus, the 3D reconstruction quantification method may be useful tools for monitoring TB treatment. The association with serum indices are not only inexpensive and sensitive but also may be incorporated into the assessment of patients during TB treatment.

## Background

Tuberculosis (TB) is an infectious disease with high rates of morbidity and mortality 1-3. It is caused by the *Mycobacterium tuberculosis* complex, which mainly affects the lungs [4]. Annually, nearly 10 million people are infected by TB, leading to 1.4 million deaths [5-7]. Its etiological agent has become increasingly resistant to drug treatment, thus requiring changes in treatment schemes [3].

In 1979, Brazil adopted a medication system for TB treatment called Scheme I (rifampicin, isoniazid, and pyrazinamide). In 2009, the National Tuberculosis Control Program in Brazil, together with the Technical Advisory Committee, made changes to Scheme I. The 2nd National Survey of Resistance to Anti-TB drugs reported higher primary resistance to isoniazid (an increase from 4.4% to 6.0%) [8]. Therefore, ethambutol was incorporated into the treatment scheme, which they denominated Scheme II (rifampicin, isoniazid, pyrazinamide, and ethambutol). Ethambutol may prevent the emergence of drug-resistant bacilli during therapy [9]. This recommendation was applied by the World Health Organization and is currently used in most countries [8]. 

Acute-phase reaction serum tests are essential for monitoring TB patients [10, 11]. The inflammatory reaction causes a characteristic increase in blood proteins [12]. Pulmonary infection with *M. tuberculosis* is also associated with increased neopterin levels [13]. C-reactive protein (CRP) levels are directly related to disease activity [14, 15]. Higher proteins levels that are caused by inflammation elevate the erythrocyte sedimentation rate (ESR) [16, 17]. Therefore, CRP and ESR levels are reliable factors for monitoring TB patients. Despite chemotherapy, patients with cured pulmonary TB may still present lung involvement and functional impairments in pulmonary function [18, 19]. Little is known about the long-term effects of TB treatment on pulmonary function [20]. Pulmonary function tests measure lung-breathing capacity, which may be related to functional damage posttreatment.

The most accurate imaging method for evaluating TB patients is high-resolution computed tomography [21]. However, this method generates high radiation doses and is associated with higher costs compared with chest radiographs [22]. X-rays are the most commonly used imaging modality for identifying patients with abnormal pulmonary status. This type of image has been useful for evaluate lung lesions even in asymptomatic patients [23]. Viewing compromised TB structures on X-ray images is difficult, and computer-aided tools can enhance detection and quantification. Giacomini *et al*. [24] developed an algorithm that is able to determine equivalence between high-resolution computed tomography and X-ray examinations, thus enabling the quantification of three-dimensional pulmonary involvement based on chest X-ray images.

The present study sought to determine the level of significance between lung involvement, serum indices, and pulmonary function during the pre- and posttreatment stages of TB. This procedure was applied to patients who received two anti-TB treatment schemes (Schemes I and II).

## Methods

### Patient sample

Patient information was acquired and analyzed in accordance with the ethics committees of the authors’ institutions. All of the patients’ files, imaging examinations, and diagnostic evaluations were retrospectively obtained between 2007 and 2014. 

This study included 50 male patients who were diagnosed with pulmonary TB, treated for 6 months, and cured posttreatment. The inclusion criteria in the patient samples were the following: patients with confirmed TB based on positive bacilloscopy or mycobacterium isolation in culture medium [25] who underwent chest radiography, serum tests and pulmonary function tests. The exclusion criteria were: the presence of systemic disease (for example hypertension and arthritis), or diseases that could compromise the lungs (bronchitis, asthma, and pneumonia), and aggravating factors (except smoking and alcoholism).

### Data collection

We evaluated 25 patients who received treatment Scheme I (rifampicin, isoniazid, and pyrazinamide) and 25 patients who received treatment Scheme II (rifampicin, isoniazid, pyrazinamide, and ethambutol). All of the patients had chest X-ray exams both pre- and posttreatment. Each exam contained two images: postero-anterior (PA) and profile (P) view. Three serum tests: levels of albumin (ALB), ESR and CRP were evaluated pre- and posttreatment. Pulmonary function tests were performed posttreatment according to the American Thoracic Society/European Respiratory Society guidelines for subject maneuver, techniques, and quality control [26].

## Protocols and analysis of serum tests and pulmonary function tests 

### Serum tests 

C-reactive protein and ALB levels were determined using Ortho-Clinical Diagnostic Equipment, System Vitros Chemistry, model 5.1 FS (Johnson & Johnson). Indices were collected using a dry tube containing 5 ml of serum that was centrifuged at 3000 rpm for 10 min. Erythrocyte sedimentation rates were determined using the manual erythrocyte sedimentation technique (i.e., aspiration of the blood sample with sedimentation observation after 60 min). The serum tests were performed pre- and posttreatment. Serum indices were compared with normal reference levels. The reference levels for these tests were: CRP < 1 mg/dl, ESR ≤ 10 mm/h, and ALB 3.5-5 g/dl [14, 27]. Serum changes were considered when the indices differed from the reference levels.

### Pulmonary function tests

Pulmonary function was assessed using a Koko Pulmonary Function Testing Model C080501 (NSpire Health Ltd). The data were obtained according to the Guidelines of Pulmonary Function Testing, with the bronchodilator applied 15 min after the first phase. The pulmonary function test measures airflow and the presence of obstructions in the airways or bronchi and determines lung volume. Evaluation of pulmonary function determined the presence of the following ventilator defects: restrictive, obstructive, or mixed. Forced vital capacity (FVC), forced expiratory volume in 1 s (FEV1), and the FEV1/FVC ratio was measured. Airflow obstruction was defined as post-bronchodilation FEV1/FVC < 70% with FVC > 80%. Restrictive defects were defined as FEV1/FVC ≥ 70% with predicted FVC < 80%. Mixed defects were defined as predicted FVC < 80% and FEV1/FVC < 70% [28]. The pulmonary function tests were also graded on a scale from 0 to 3, based on the severity of the functional disorder: 0 = normal, 1 = mild, 2 = moderate, 3 = severe.

## Protocol and analysis of Computed Radiography image quantification of lung involvement

### Protocol for image acquisition 

Chest X-ray examinations were performed using the following parameters: 1.80-meter film source distance, 90-120 KVp, 2-8 mAS, 43 cm ( 35 cm image plate size, and profile (P) and postero-anterior (PA) projections.

### Image analysis and quantification of lung involvement

Image analysis and quantification of lung involvement: We used a computational algorithm developed by Giacomini et al. [24]. This approach quantifies lung involvement based on CR images. The algorithm consisted of the following:

Step 1. Chest radiograph segmentation. Computed radiography (CR) images in the PA and P views were loaded and read. The regions of interest were manually segmented by a radiologist who selected the lung area for analysis.

Step 2. Binarization and expansion. The defined lung areas were binarized and expanded.

Step 3. Lung volume. The total lung volume was based on intersections between the expansions in both views.

Step 4. Lung involvement. The manual segmentation of involvement in the X-ray PA projection was determined. The signal-difference-to-noise ratio (SDNR) of the compromised lung region was calculated, and the thickness of lung involvement was estimated [24].

**Figure 1:**
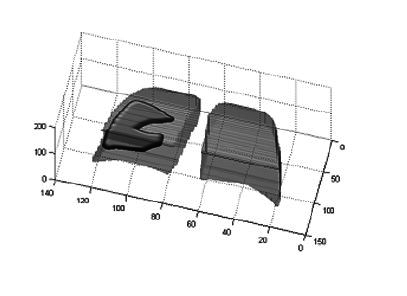
Representation of compromised lung volume in three-dimensional view.

Step 5. Volumetric quantification. The quantification of three-dimensional pulmonary involvement was determined. This impairment was estimated by counting the pixels that belonged to the lungs and affected regions (Fig. 1). This objective quantification was performed both pre- and posttreatment.

## Analytical methodology

Serum exams and the quantification of lung involvement were compared pre- and posttreatment between Schemes I and II. We also compared the pulmonary function test results with the algorithm-based quantification for the two schemes. We also evaluated the reduction of lung involvement (RLI) according to the following equation:

$$RLI\%=\left|\left(Vf-Vi\right)/Vi\right|*100$$

where Vf is the objective quantification of lung involvement posttreatment, and Vi is the objective quantification of lung involvement pretreatment.

## Statistical analysis

We reported the means and standard deviations of normally distributed values, and the interquartile ranges of non-normally distributed values. Mood median test with p = 0.003 was performed to compare the values pre - and posttreatment. Statistical analysis by Mann Whitney with p = 0.05 was performed to compare Schemes I and II, and clinical data. The Pearson correlation was applied to correlate a three-dimensional computed radiography (3D) method and pulmonary function tests. The assessment from reduction lung involvement by the 3D method and serum indices were compared using Bland-Altman statistics to assess agreement between methods [29], quantify the amount and direction of bias, and determine the upper and lower limits of agreement (bias ± two standard deviations).

## Results

We characterized the clinical data from all patients (Table 1). This procedure was applied to control for similarities and differences among the patient samples. No significant differences in age, symptom duration, or smoking history were found between the two patient groups (p > 0.05, Mann-Whitney U test).

**Table 1:** Comparison of clinical data from TB patients who were treated with Scheme I or Scheme II. The data are expressed as mean ± standard deviation showing significant similarities between the used patient samples.

Clinical data	Scheme I	Scheme II	p*
Age (years)	58 ± 16	56 ± 12	0.71
Symptom duration (months)	3 ± 3	2 ± 1	0.83
Smoking history (packs-per-day years)	52 ± 43	50 ± 35	0.94

* Statistical analysis results: Mann-Whitney U test, p > 0.05.

**Figure 2:**
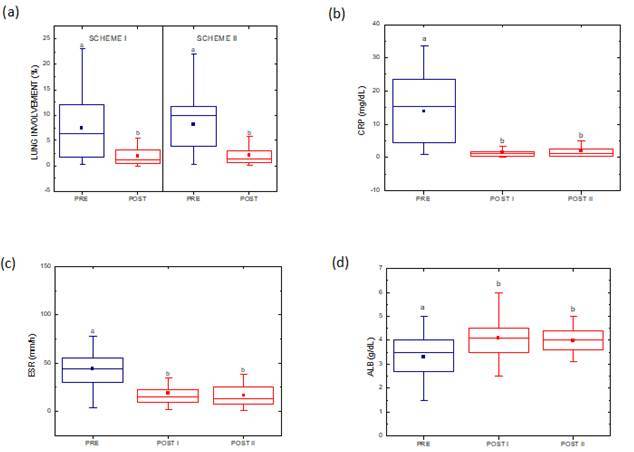
(a) Analysis of lung involvement between Scheme I and Scheme II (pretreatment median = 6% for Scheme I; pretreatment median = 10% for Scheme II; posttreatment median = 1% for both Schemes I and II). (b) Box-plot of CRP pre- and posttreatment in patients who were treated with Scheme I or Scheme II (POST I and POST II, respectively). Schemes I and II: pretreatment median = 15 mg/dl, posttreatment median = 1 mg/dl. (c) Box-plot of ESR pre- and posttreatment in patients who were treated with Scheme I or Scheme II (POST I and POST II, respectively). Scheme I and II: pretreatment median = 44 mm/h. Scheme I: posttreatment median = 15 mm/h. Scheme II: posttreatment median = 13 mm/h. (d) Albumin levels increased posttreatment compared with pretreatment in patients who were treated with Schemes I and II. The lower and upper boundaries of the boxes indicate the 25th and 75th percentiles, respectively. The solid horizontal lines in the boxes indicate the median. The squares represent the mean. The whiskers above and below the boxes represent the maximum and minimum values, respectively. ^a, b^, significant difference (statistical analysis by Mood median test with p < 0.003 between pre- and posttreatment; and statistical analysis by Mann Whitney with p > 0.05 between Schemes I and II).

The lung-involvement percentage and the levels of CRP, ESR and ALB were determined pre- and posttreatment (Figure 2). Lung involvement decreased posttreatment (2% ± 2%) compared with pretreatment (8% ± 6%; Mood median test, p = 0.003; Fig. 2.a). C-reactive protein and ESR levels decreased posttreatment compared with pretreatment (Fig. 2.b and 2.c). Albumin levels increased posttreatment compared with pretreatment (Mood median test, p = 0.003; Fig. 2.d). Scheme I and Scheme II treatment did not interfere in the lung involvement parameter. The two groups had presented similar responses, namely decreasing the initial involvement.

## Evaluation of reduction of lung involvement based on X-ray images

Based on the objective quantification of lung involvement, we measured the RLI% in patients who were treated with Schemes I and II. The patients treated with Scheme I had 73±12% reduction of lung involvement. The patients treated with Scheme II had a 70±17% reduction. The difference in the treatment schemes did not interfere in a reduction of lung involvement. The RLI% decreases did not differ statistically between the two treatment schemes (Mann Whitney, p > 0.05).

## Determination of lung involvement based on X-ray images and serum test results

**Figure 3:**
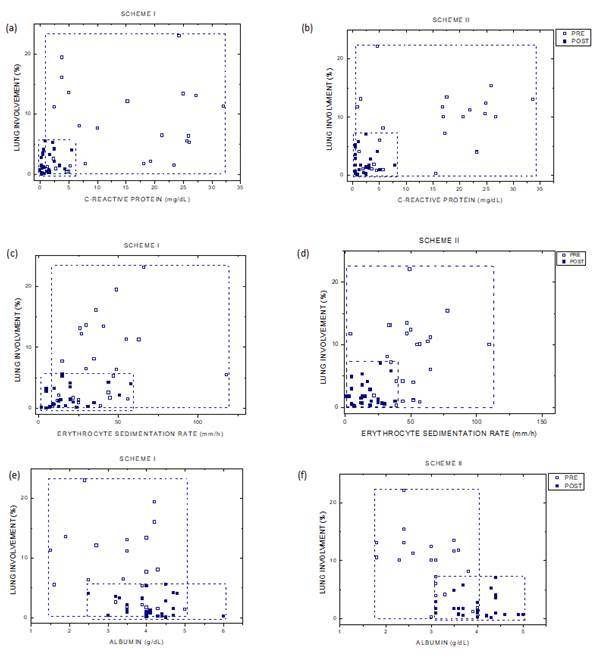
Panel of patient maps and serum index - Ordinate in the plots represents the lung-involvement percentage and abscissa the correspondent serum index. The first panel line shows the lung-involvement percentage versus the C-reactive protein levels for Scheme I (a) and Scheme II (b). The second panel line displays the lung-involvement percentage versus erythrocyte sedimentation rate for Scheme I (c) and Scheme II (d). Third line is the lung-involvement percentage versus the albumin levels for Scheme I (e) and Scheme II (f). Statistical analysis by Mann Whitney with p > 0.05 between Schemes I and II. Open square (full square) represent patients results pre- (post-) treatment. As expected, a clear reduction of sample area was observed posttreatment, with serum indices within the normal range of values. The plots show that the novel 3D reconstruction method of evaluate lung involvement accompanies the reduction in the serum indices.

Based on comparisons of the serum test results and lung involvement, we correlated the image quantification of pulmonary involvement with CRP levels, ESR, and ALB levels (acute-phase reaction). Figure 3 shows the mapping of samples between lung involvement and serum indices pre- and posttreatment in patients who received Schemes I and II. We observed a reduction in the area of dispersion between the correlations pre- and posttreatment. Both groups (Scheme I and Scheme II) had recovered similarly (Mann Whitney, p > 0.05). 

**Figure 4:**
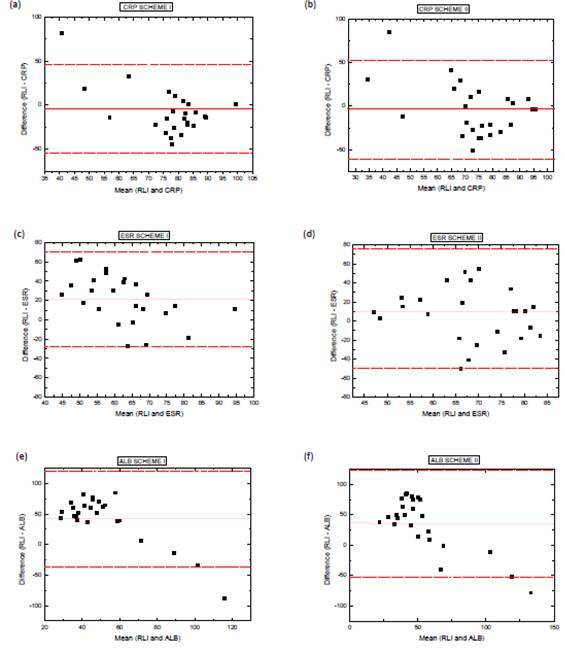
Bland-Altman plots for scores of: A) RLI and CRP for Scheme I; B) RLI and CRP for Scheme II; C) RLI and ESR for Scheme I; D) RLI and ESR for Scheme II; E) RLI and ALB for Scheme I; and F) RLI and ESR for Scheme II. The difference between methods was compared with the average between methods. Short dashed lines indicate the interval of two standard deviations, indicating an excellent level of statistical agreement between the results.

Figure 4 displays the Bland-Altman plots of the score difference between the lung-involvement reduction by 3D method and serum indices. The methods did not differ statistically; through the evaluations we found agreement between RLI and serum indices. 

## Lung involvement based on X-ray examination vs. pulmonary function tests

In the pulmonary function tests, obstructive defects were the most common ventilator disorder (48%). Restrictive defects and normal function were present in 22% and 16% of the patients, respectively. Mixed defects were present in 15% of the patients (Table 2). A high correlation was found between the degree of pulmonary dysfunction and quantification of lung involvement (r=0.87, p=0.33 Scheme I; r=0.98, p=0.02 Scheme II). Degree 1 was the most common level of severity in patients who received Scheme I (35%), while Scheme II presented the same frequency for different severity degrees (27%).

**Table 2:** Pulmonary function tests and severity of pulmonary dysfunction in patients who were treated with Schemes I and II. Lung damage is represented as a mean.

		Frequency in Scheme I	Lung involvement in Scheme I	Frequency in Scheme II	Lung involvement in Scheme II
Pulmonary function test	Obstructive defects	13/25 (52%)	1.3%	11/25 (44%)	2.3%
	Restrictive defects	6/25 (24%)	3.3%	5/25(20%)	2.7%
	Mixed defects	4/25 (16%)	2.9%	3/25 (12%)	1.0%
	Normal	2/25 (8%)	0.2%	6/25 (24%)	1.8%
Severity of pulmonary dysfunction	0	2/21 (10%)	0.2%	6/22 (27%)	1.8%
	1	7/21 (35%)	0.4%	6/22(27%)	0.9%
	2	6/21 (30%)	1.8%	4/22 (19%)	1.3%
	3	5/21 (25%)	3.5%	6/22 (27%)	4.6%

Note: Patients who had mixed defects in pulmonary function tests are not included in the analysis of pulmonary dysfunction severity.

## Discussion

The present study analyzed patients with pulmonary TB who had received two different treatment schemes. We then determined associations between lung involvement, serum indices and the results of pulmonary function tests. The patient groups had similar characteristics with regard to age, symptom duration and smoking history. Smoking is an important factor that significantly influences the development of TB [30]. Willcox and Ferguson showed that smoking had an additive effect on airway obstruction [20]. However, all patients who were evaluated in the study presented a statistically similar smoking load, which implies similarity of possible pulmonary impairment.

We quantified lung involvement based on exams pre- and posttreatment using a three-dimensional analytical method [24]. The quantification of pulmonary involvement confirmed statistically similar reductions posttreatment under the two schemes (Mood median test, p = 0.003; see figure 2.a). The RLI% decreases did not differ statistically between the two treatment schemes (Mann Whitney, p > 0.05).

Some studies have reported adverse reactions to anti-TB drugs with different degrees of severity [31]. In this study, we used two different treatment schemes. With the continual increase in the bacterial resistance to drugs, the incorporation of ethambutol in the treatments is necessary [8]. Bobrowitz et al. [32] showed that the use of ethambutol was the best tolerated regimen and provided adequate treatment for advanced tuberculosis. However, our findings with regard to lung involvement pre- and posttreatment indicated that both schemes led to a similar recovery of lung function and pulmonary involvement. Moreover, no results indicated that ethambutol interferes in the degree of the final lung injury when compared to other treatments. In an effort to overcome resistance to isoniazid, ethambutol retained its efficacy in inhibiting mycobacteria and did not affect the sequelae of lung involvement. 

The serum tests of CRP and ESR indicated the existence of a pretreatment inflammatory process that was attenuated by both treatment schemes (see fig 2.b and c). High pretreatment CRP and ESR levels in both groups of patients were directly related to the degrees of inflammation and disease activity [12], presenting high lung involvement. We found significant differences in CRP and ESR levels between pre- and posttreatment in the two groups (Mood median test, p < 0.001), with no differences between Schemes I and II (Mann Whitney, p > 0.05). Albumin is a negative acute-phase protein, which decreases during inflammation and can also be used to assess nutritional status [33]. Our pretreatment findings revealed low ALB levels, suggesting malnutrition in the patients. Posttreatment, ALB levels increased to reference levels, indicating an improvement in the patients’ nutritional status (see Fig 2.d).

The maps in Figure 3 indicate that lung involvements were directly related to the serum test results, reflected by similar reductions of lung involvement between pre- and posttreatment. The serum tests were an excellent tool for monitoring the efficacy of TB treatment, and both Scheme I and II treatments resulted in a tendency of the serum indices to approach reference levels. The results for mean values of pre- and posttreatment are strongly affected by heterogeneity in the patient sample. Figure 3 shows a high reduction of lung-involvement volume in Schemes I and II, bringing serum indices to normal range. Our results displayed in Figure 4 indicate significant agreement between 3D method and serum indices. This concordance suggests that the 3D reconstruction method can be employed to monitor tuberculosis, since it agreed closely with the acute-phase serum indices, contributing to a new analysis parameter related to TB sequelae. 

Even in the event of a microbiological cure, other variables should be considered when evaluating sequelae in patients. Some posttreatment consequences involve functional lung impairment and lung damage [19]. Previous studies reported that more than half of patients who complete treatment have subsequent pulmonary impairment [28]. Long et al. [34] found that restrictive lung defects were more prevalent in patients with cavitary areas. Restrictive lung defects were also reported by Candela at al. [35] and Mbatchou Ngahane et al. [28]. The destruction of lung parenchyma has been a characteristic effect of restrictive disorders [18]. However, the predominance of obstructive lung defects in the present study (48% of patients) corroborated the results of Willcox and Ferguson [20] and of Lee and Chang [36].

The obstructive disorder was prevalent in the present study, which is caused by partial deterioration of the bronchial walls that leads to the loss of radial traction and consequently airflow impairment [18]. Chung et al. [37] reported that bronchial involvement is an important predictor of the deterioration of pulmonary function and the presence of obstructive disorders after treatment is completed. Notably, normal exams were observed three times more frequently in patients who were treated with Scheme II (24%) compared with Scheme I (see Table 2). However, the objective quantification of lung involvement in patients who had normal pulmonary function presented high variability, with no significant differences from other pulmonary disturbances (Mann-Whitney, p > 0.05).

We also associated the degree of severity of functional dysfunction with the objective quantification of impairment. Larger pulmonary lesions may cause greater functional lung damage. Our results corroborated Chung et al. [37], who found that the subjective radiological extension of TB on X-ray images was associated with functional lung involvement. Plit et al. [38] reported similar results, namely that lung impairment assessed by subjective radiological scores influenced posttreatment lung function. Table 2 shows that the evaluation of lung involvement based on 3D reconstruction images was correlated with the pulmonary function tests. 

## Conclusion

We applied the 3D reconstruction method to assess lung involvement in a cohort of treated TB patients and compared the results to serum indices and pulmonary function tests. We also found a high coincidence level of results mainly between the 3D reconstruction method with C-reactive protein and the degree of functional lung impairment posttreatment. The methodology applied herein does not require any additional procedures or new examinations in patients with TB, and provides a new approach and contributes to a better evaluation of the patient’s condition. 

### Abbreviations

ALB: albumin; CR: computed radiography; CRP: C-reactive protein; ESR: erythrocyte sedimentation rate; FEV: forced expiratory volume; FVC: forced vital capacity; P: profile; PA: postero-anterior; RLI: reduction of lung involvement; SDNR: signal difference-to-noise ratio; TB: tuberculosis; V_f_: objective quantification of lung involvement posttreatment; V_i_: objective quantification of lung involvement pretreatment.
